# The Effects of Massage Therapy on Multiple Sclerosis Patients' Quality of Life and Leg Function

**DOI:** 10.1155/2014/640916

**Published:** 2014-05-08

**Authors:** Brittany Schroeder, Jennifer Doig, Kalyani Premkumar

**Affiliations:** College of Medicine, University of Saskatchewan, HSc 3226 E Wing, Clinic Place, Saskatoon, SK, Canada S7N 5E5

## Abstract

*Background*. Massage therapy is a noninvasive treatment that many individuals with multiple sclerosis (MS) use to supplement their conventional treatment. *Objective*. We hypothesize that massage therapy will improve the leg function and overall quality of life (QoL) of MS patients. *Design*. A two-period (rest, massage) crossover design was used. Twenty-four individuals with MS ranging from 3.0 to 7.0 on the Expanded Disability Status Scale (EDSS) received Swedish massage treatments for four weeks. Exercise capacity and leg function as well as QoL were assessed using the Six-Minute Walk Test (6MWT) and the Hamburg Quality of Life in Multiple Sclerosis (HAQUAMS) instrument, respectively. Assessments were measured before and after a massage period and a rest period where no massages were employed. *Results*. The results displayed no significant changes in 6MWT distances or HAQUAMS scores. However, the participants perceived improvement in overall health as expressed in written comments. *Conclusions*. Massage is a safe, noninvasive treatment that may assist MS patients in managing the stress of their symptoms. Future studies with larger sample size and cortisol measures are warranted.

## 1. Introduction

Multiple sclerosis (MS) is a demyelinating disorder of the upper motor neurons. It is estimated that over 100,000 Canadians suffer from the aftermath of this neurological disorder with the prevalence ranging from 1 in 500 to 1 in 1000 people in different parts of the country [1]. Degeneration of neuronal myelin sheath results in abnormal firing of the upper motor neurons. This leads to a variety of debilitating symptoms including hypertonicity of the muscles known as spasticity, which can cause pain and affect mobility. Pain is a direct result of activation of mechanosensitive pain receptors in the muscle as well as the ischemia from compression of the microcirculation [2].

Chronic stress has been implicated in MS progression as it can weaken cytokine response, which further aggravates symptoms [3]. Short episodes of stress activate the hypothalamic-pituitary-axis (HPA) which releases cortisol in order to regulate inflammation. Chronic stress, such as that experienced in a chronic illness, causes a prolonged cortisol release, which, over time, causes a downregulation of glucocorticoid receptors. This in turn decreases the HPA's capacity to fight inflammation [3]. Regulation of stress and the use of relaxation techniques are important in symptom management of MS, an inflammatory illness.

The majority of current treatments for spasticity and “nerve pain” are pharmaceutical. However, due to individual variability in the presentation of symptoms, MS sufferers are often left dissatisfied and therefore seek relief through alternative treatments. Massage therapy is one of most common complementary and alternative medical (CAM) practices used by MS patients [4].

Relaxation such as that promoted by massage therapy allows an individual to enter into a lower anxiety state by increasing parasympathetic signals [5]. Preoperative massage treatments have been shown to reduce pain, anxiety, and tension in patients undergoing cardiac surgery [6]. Promoting relaxation increases parasympathetic signalling which in turn would help to decrease an elevated cortisol release. This could have a profound impact on MS symptom management and quality of life experienced by the individual. Improvements have been observed in the mental and emotional state of MS individuals after receiving massage treatments [7].

Although the exact mechanisms for these positive responses are understudied in the literature, there are several prevalent theories. The tension of a tight muscle can directly cause pain by activating mechanosensitive pain receptors in the muscles as well as restricting blood flow to cause acute, local ischemia. The strokes and friction applied during massage are thought to promote muscle and connective tissue fibers to relax. This would allow the fibers to release their tight association with neighbouring fibres and to be realigned properly. In turn, the pain receptors would no longer be activated and blood flow would be promoted [2].

The pressure of therapeutic touch also provides a short-lived analgesic effect through the activation of subcutaneous mechanoreceptors. Once activated, these receptors block the signals from the pain receptors that arrive at the same spinal segment [8, 9]. Another theory explains that the light touch of Swedish massage transiently reinforces an individual's existing discomfort and thus triggers a greater release of natural opiates such as *β*-endorphins. This in turn would mediate a more profound pain suppression which would promote greater ease of movement [10].

Currently the available literature does not strongly support the theories of massage therapy enhancing muscle performance. However, it may be noted that most research is performed on professional athletes after maximal exercise or after injury, making it difficult to generalize findings to spasticity in MS patients. In addition, most of these studies measure the immediate effects of massage on performance. Our study focuses on the functional effects after several weeks of massage treatments.

There has been no research conducted on the functional effects of massage therapy in MS prior to this study. We hope to observe any changes in walking capacity as an indication of leg function. In addition, we will add further knowledge to the QoL improvements experienced by individuals as a result of massage treatments. We believe that the positive benefits offered through massage therapy will greatly assist MS individuals in their symptom management. We anticipate that the level of spasticity experienced by individuals will be lessened as a direct result of the massage treatments, which in turn may promote improvements to their walking capacity. We hypothesize that the benefits of massage therapy will promote an improvement to the overall QoL experienced by MS individuals.

## 2. Methods

### 2.1. Participants

Participants were recruited via posters displayed at the three hospitals in Saskatoon, SK, and at the MS society office in Saskatoon, SK, as well as through newspaper advertisements. Fifty-seven volunteers were interviewed; 31 patients were turned away due to their lack of availability or failure to meet the patient criteria. The data from 2 patients was removed due to the absence of more than 3 massage treatments. The final 24 participants met our criteria of disability, which are defined as a score between 3.5 and 7.0 on the Expanded Disability Status Scale (EDSS), a score that grades the quantum of disease burden. Data on type of MS was not collected as the focus of this study was on the effect of massage on symptom management. Each participant committed to receiving no massages outside of what was included in the study for the duration of the 8 weeks. Patients were asked to make no other changes to their current treatment plans and to inform the research team if and when changes occurred such as the introduction of a new medication.

*Inclusion/Exclusion Criteria*. Multiple sclerosis patients rated between 3.0 and 7.0 on the self-administered Kurtzke Expanded Disability Status Scale (self-EDSS) at the time of recruitment were recruited. The group was not gender or age specific. For the purpose of measuring change in MS patients' ability or rather in their disability, there needs to be some visible, measurable dysfunction present. Thus, any stages lower than 3.0 on the EDSS scale would not be sufficiently testable. In addition, due to certain restrictions of this particular study, MS patients need to be able to get themselves to and from the massage clinic in order to participate and so the severity level of MS is capped at 7.0 to ensure sufficient mobility. Therefore, EDSS scores below 3.0 and above 7.0 were not accepted for participation. Exclusion criteria also included chest pain (unstable angina) and/or a heart attack (myocardial infarction) during the previous month, high blood pressure (blood pressure of 180 mmHg systolic over 100 mmHg diastolic), and inability to attend regular massage appointments and assessments on the 3rd floor of a building (elevator access available). The above restrictions regarding heart conditions are guidelines set by the American Thoracic Society for the Six-Minute Walk Test, which will be conducted during pre- and postmassages periods.

### 2.2. Ethics

The University of Saskatchewan's Behavioural Research Ethics Board approved this study. Written consent was obtained from all participants prior to the start of the study. This included consent to publish data in an aggregate form in scientific journals.

### 2.3. Study Design

A two-period crossover design was chosen to enable a comparative analysis between treatment and nontreatment groups. Each patient received 2 massages per week for 4 weeks. Assessments were conducted before and after the treatment intervention periods in order to observe any changes.

**Figure 1:**
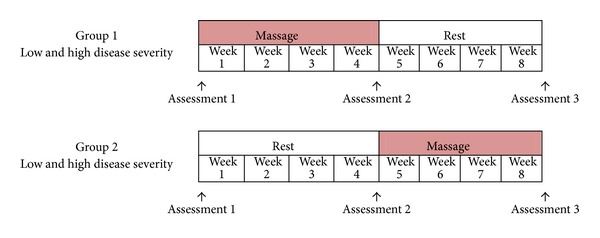
Two-period crossover study design. Group 1 received massages from weeks 1 to 4. Group 2 receives massages from weeks 5 to 8.

Each participant was randomly assigned into one of two groups. Group 1 received massages in the first four weeks of the study while group 2 received no massages. During the last four weeks of the study, group 2 received massages while group 1 received no massages (Figure 1).

Each patient received the same standardized massage routine upon each visit. Prior to the study, the massage therapists were trained by a supervisor to maintain the standard routine and the treatment routine was posted at the head of the massage table as reminder.

The massage treatment was a relaxing 45-minute whole-body Swedish massage with focus on the lower extremities. The posterior lower limbs were massaged for 15 minutes with the patient in prone position and 10 minutes were allotted to massaging the anterior parts of the lower limbs. The strokes included compressions, effleurage, deep palmer stroking, C-scooping, picking up, thumb kneading, forearm stripping, wringing, and light stroking and were delivered in the same order for each part of the body. Two massage therapists were assigned to each patient in order to eliminate bias in massage delivery and thus to ensure a similar experience for all patients.

### 2.4. Assessments

#### 2.4.1. Immediately after Each Massage Treatment

- Patient 30-second massage comparative questionnaire: to observe the degree of similarity between each treatment experienced.
- Massage therapist questionnaire: regarding the duration of treatment and any observed changes in patients.

#### 2.4.2. Start and End of Massage and Rest Periods (Assessment 1, Assessment 2, and Assessment 3)

- Six-Minute Walk Test (6MWT): to observe exercise capacity as a secondary measure of leg function.
- Hamburg Quality of Life Questionnaire in Multiple Sclerosis (HAQUAMS): it consists of subsections involving different aspects of physical, mental, emotional, and social health.
- Expanded Disability Status Scale (EDSS): to measure level of disability.

At assessment 1, a brief medical history was taken for each patient. At assessment 3, a final comments questionnaire was administered.

#### 2.4.3. Weekly Assessments

 Weekly three-question health assessment: to log any stressors that may have aggravated patient MS symptoms.

### 2.5. Assessment of Leg Function

The 6MWT provided a secondary approach to observe leg function as it is a reliable and valid measure of exercise capacity in MS [11]. This test was chosen as it is easy to measure and is a reflection of activities of daily living.

The 6MWT course consisted of a pylon at each end of a 25-metre stretch of a straight, empty hallway. Participants were instructed to walk at a casual and comfortable pace towards the pylon at the end of the hallway without resting for 6 minutes. A demonstration was provided for each patient before each walk test as well as an explanation that patient and technician were asked to refrain from speaking during the test. Because individuals, especially those with muscular dysfunctions, rarely walk intensely for 6 minutes without stopping, at the first assessment the 6MWT was conducted two times, one hour apart, in order to rule out learning and anxiety variables. The above guidelines have been determined by the American Thoracic Society [11].

### 2.6. Assessment of Quality of Life

The Hamburg Quality of Life in Multiple Sclerosis (HAQUAMS) instrument is divided into five subcategories: fatigue and cognitive function (four items), lower limb mobility (five items), upper limb mobility (five items), social function (six items), and mood (eight items). The quality of life of an individual may be assessed by calculating the average score of the five subscales, weighing each subscale evenly. In addition, the subgroups may be assessed individually. HAQUAMS offers application to a wide range of disease statuses such as that seen in this study and is a reliable and valid instrument for QoL assessment in multiple sclerosis [12].

The final question of the HAQUAMS allows participants to define the state of their health and disability. This question was teased out, assessed, and referred to as “Personal Health Rating” in this paper.

### 2.7. Statistical Analysis

Paired sample *t*-tests were carried out to observe changes to 6MWT distances and the HAQUAMS averages after the massage period as well as after the rest period. Independent *t*-tests were also used to compare the outcomes of the two groups and the two disability levels. In order to look at changes in individuals, each patient served as their own control. All calculations were performed at a 95% confidence interval using SPSS 16.0 software.

After data collection, each group was further divided according to ambulation-defined disease severity groups according to the Kurtzke EDSS. Higher severity is defined as an EDSS score of 5.0 and higher, which is indicative of impairment to ambulation [4]. Therefore, lower severity in this study was defined as 3.0–4.9.

Repeated-measures ANOVAs were conducted where measures at each of the three time points were entered as within-subject factors and MS severity and condition were entered as between-subject factors. When a main effect or interaction was found, post hoc *t*-tests were conducted to identify where the difference was. Effect sizes (Cohen's *d*) were also calculated as a measure of practical significance.

## 3. Results

The characteristics of the patients who participated in the study are given in Table 1.

### 3.1. Patient 30-Second Massage Comparative Questionnaire and Massage Therapists Questionnaire

There was an overall consistency of massage treatments delivered according to the questionnaires and the differences between massage treatments were not of significance.

There were no noteworthy differences in massage treatments delivered throughout the study according to the massage therapist questionnaire.

### 3.2. 6MWT

A main effect of distance was found; *F*(2, 40) = 3.94, *P* = .027, and *d* = .12. However, no significant interactions between distance and severity or condition were found. Post hoc *t*-tests for distance indicated that all, overall, participants were able to walk further at assessment 2 (M = 405.67, SD = 155.96) than assessment 1 (M = 387.42, SD = 154.10), regardless of disease severity or condition; *t*(23) = −2.74, *P* = .011, and *d* = −.12.

Means, standard deviations, and effect sizes for each group and severity condition are reported in Table 2.

### 3.3. HAQUAMS

Lower scores on the Hamburg represent a more positive quality of life status. Negative changes observed in Table 3 indicate an increase in quality of life status.

Independent *t*-tests using group 2 during their rest period as the control showed no significant differences in changes to the control group HAQUAMS score compared to either massage group.

Age and gender had no impact on the differences in group performances as each group had similar demographic distribution. The greatest improvements were observed in group 1 higher disease severity individuals after massage treatments. There were no statistically significant main effects or interactions between HAQUAMS average score, severity, and condition. Means, standard deviations, and effect sizes for each group and severity condition are reported in Table 3.

### 3.4. Personal Health Rating

There was a statistically significant interaction between personal health rating and condition; *F*(2,40) = 4.51 and *P* = .017. Post hoc *t*-tests revealed that group 2 significantly improved after massage treatment; *t*(10) = 2.67, *P* = .024, and *d* = 1.02. This difference yielded a large effect size. Means, standard deviations, and effect sizes for each group and severity condition are reported in Table 4.

### 3.5. Self-Assessed Expanded Disability Status Scale (EDSS)

A statistically significant interaction between personal health rating, condition, and severity was found; *F*(2,40) = 3.29 and *P* = .047. Post hoc *t*-tests did not reveal any statistically significant differences.

### 3.6. Patient Comments

There were 18 positive and 3 negative comments regarding the effects of massage on the lower legs. Out of the 18 positive comments, 5 patients noted that the benefit lasted several hours after the massage treatments while 13 patients noted that massage had a positive impact on their legs that lasted several days to weeks after the massage treatments.

When asked if the massage treatments were relaxing, 23 patients responded with yes and 1 patient replied with no. Six patients noted that the massages helped them sleep better at night and 3 patients noted an increased energy level the next day.

### 3.7. Weekly 30-Second Questionnaire

Overall, there were no changes in self-reported health status.

## 4. Discussion

### 4.1. Summary

Overall, massage did not significantly change the 6MWT distance or the HAQUAMS scores of study participants. However, the walk distances of group 1 patients and HAQUAMS scores of group 1 patients with a higher disease severity significantly improved after massage treatments. In addition, after massage treatments, both groups displayed an overall improvement in their personal health rating. When massage was removed from group 1, there was depreciation in their personal health rating. Analysis of patient comments supports these findings.

### 4.2. 6MWT

The unchanged walking capacity of group 2 and the improvement observed in group 1 walk distance after massage treatments suggest that massage was not causing harm and it has the potential to improve mobility in certain individuals.

The improvements observed could have been due to the restoration of proper muscle mechanics. Many individuals began the study with tight muscles. Structurally, these cramped, shortened muscles have a higher degree of overlap of actin and myosin fibrils than normal, which functionally limits their strength and ability to contract [13]. A common characteristic in MS is a condition called foot drop which is the result of an over-taut soleus tendon that holds the foot in a forced plantar flexion [14]. This greatly interferes with mobility as it forces the individual to lift her leg higher than normal in order to take each step. Muscle tightness and pain often result in both the spastic muscles and the opposite leg which works harder to compensate for the dragging foot. Massage may have relieved the tightness and pain in the surrounding muscles that were being overworked as well as in the spastic muscle. Massage strokes stretch the muscle and provide heat and friction to loosen adhering muscle fibres. This forces the tightly overlapped microfilaments to release and be realigned properly which allows for better muscle function and thus partial restoration of mobility [2].

These improvements observed in walking capacity are supported by studies conducted on the acute effects of massage and exercise performance. When Monedero and Donne compared the effects on performance recovery in male cyclists after maximum exercise, they found that a combined treatment of massage and active recovery was the most efficient intervention when compared to either passive, active, or massage regimes alone [15]. Another study measured the number of leg extensions that healthy subjects could perform after a period of intense exercise followed by either massage or merely rest. Individuals receiving massage displayed an increased number of leg extensions indicating an improved performance in quadriceps muscles [16]. However, it should be noted that, according to a review on performance and muscle recovery in athletes and massage intervention, results were mixed and unreliable due to small sample sizes and inconsistent methodologies [2].

Restoration in function may also be due to a decrease in pain. As mentioned earlier, tight and cramped muscles cause pain by applying pressure on mechanosensitive pain receptors as well as by blocking microcirculation. By reducing tension in the muscles, massage directly relieves pain, which in turn improves movement.

For some individuals with MS, an increased muscle tone in the leg or back muscles can compensate for the muscle weakness also experienced in MS. Thus, a certain degree of spasticity present in some MS individuals actually assists in their balance. Therefore caution is required when treating spasticity in MS. The muscle relaxation brought about in these individuals could create a further impairment to their mobility. However, our data reveals that massage did not deter the walking capacity and leg function and only one patient noted a slight increase in muscle weakness after massage. This further proves the safety and benefit of massage therapy in MS.

### 4.3. HAQUAMS

The lack of change in the HAQUAMS total sums indicates that the massage treatment did not improve the overall health status of individuals and neither was it doing any harm. The greatest improvements were observed in HAQUAMS total sum in group 1 higher disease severity individuals after massage treatments which may simply be a result of a larger margin available for improvement. The decrease in personal health rating exhibited in group 1 individuals after massage was removed could be indicative that the effects of massage are not long-lasting. In order for individuals to continue receiving benefits from massage, the treatments would need to be continued over time at the discretion of the patient and therapist.

The improvements in personal health perception after massage could be due to an overall decreased level of pain experienced by MS individuals. As explained earlier, massage alleviates pain by relieving tension on the pain receptors, by activating mechanoreceptors to further block pain signals from ascending to the brain, and by inducing a greater release of endorphins [2, 8, 10]. In addition, it is believed by some researchers that the higher degree of serotonin released during massage has a part to play. Serotonin is involved with structures such as the raphe nuclei allowing them to inhibit pain signals from descending into the dorsal spinal column [17]. This effect could assist symptom management in MS individuals. In addition, serotonin has also been shown to have positive effects on mood, sleep, and anxiety, all of which can have an effect on an individual's experience of pain [17].

Improvement to individuals' personal health rating following massage treatments is a trend that is supported in the literature. In one study, after five weeks of massage treatments, MS patients displayed decreased anxiety, overall improved mood, a more positive opinion of the treatment management, better self-esteem, and improved body satisfaction [7]. Another study also noted a significant increase in MS patients' general health and well-being after six weeks of reflexology treatments [18].

It is also possible that the improvements in health perception were due to the benefits of relaxation and stress relief. In another study, chronic pain patients displayed 36% decreased clinic visits after behavioural medical interventions were employed to promote relaxation and decrease stress [19]. As in many chronic illnesses, stress has been implicated in the aggravation of symptoms [3]. The excess cortisol release during stress is extremely detrimental in MS, as myelin degeneration causes inflammation. In addition, chronic tension may inhibit the body from entering into a low anxiety state required for rest and repair [5]. Thus, stress management in MS protects the body's ability to control inflammation and to better manage symptoms.

A review article of research on cortisol and serotonin levels after massage treatments in the chronically ill revealed a widespread decrease in cortisol levels as well as an overall increase in serotonin levels [20]. In our study, all but one patient reported that the massages were relaxing and 18 patients reported that this relaxation lasted several hours to days after the massage treatments. This result is supported by past studies on normotensive females undergoing slow-stroke back massage. In comparison to a control group not receiving massages, a group of healthy females received a slow-stroke back massage and displayed decreased anxiety and overall relaxation prior to a written examination [5]. In another study amateur boxers during training sessions displayed a relief of tension and fatigue after massage treatments compared to control groups that merely lay resting or controls that were simply touched and not massaged [21]. Relaxation and decreased cortisol levels were also found after MS individuals received six weeks of reflexology treatments [18].

Although the HAQUAMS total sums that were collected in this study did not display significant changes after massage, the personal health rating and past research reveal that massage provides relaxation and decreases stress. In addition, the literature provides many examples of massage therapy inducing relaxation and psychological improvement. These benefits are of great importance in MS symptom management and the quality of life experienced and thus further investigation is required.

### 4.4. Limitations

One of the limitations is the small sample size. Few statistically significant differences were found. This was likely partial due to the small sample size. Several medium effect sizes were noted. Sample size calculations using G-Power indicate that a total sample size of 70 is required for a medium effect size (*f*  .25 which corresponds with *d* = .50) to be statistically significant for a repeated-measures ANOVA.

Our research, unlike those in the literature that primarily study acute effects of massage, looked at the long-term effects, which increases the difficulty in recording and comparing direct outcomes.

It should be noted that the 6MWT has been used previously to assess the exercise capacity of MS individuals in comparison to healthy individuals. However, it has not been used to measure change after a treatment intervention in MS and therefore the sensitivity of this modality is unknown. Future studies with larger sample size may consider using this test along with other leg function tests and cortisol measurements in order to obtain a more thorough assessment.

### 4.5. Future Research

Massage appeared to induce a greater effect on the quality of life in individuals with higher disease severity. Further studies are required to identify the effects of massage treatment on individuals with varying severity of disease.

There was a higher percentage of regular massage users in group 1 than there was in group 2 (58%, 18%). It may be possible that individuals in group 2 may not have been able to relax to the same degree as group 1 individuals due to the novelty of the experience. Future research would benefit by observing if previous massage experience enhances or dampens the effects of massage. In addition, the application of a “prestudy washout” period may be employed in future studies to attempt to wash out any benefits of previous massage treatments that might affect initial measurements.

### 4.6. Clinical Implications

Swedish massage is a safe, noninvasive therapy that individuals with MS could add to their current treatment regimes. Although the results from this study do not indicate significant improvements to walking capacity and leg function, patients' perception of their health improved after this relaxation therapy. This is a beneficial outcome especially in terms of stress management and overall quality of life.

## 5. Conclusion

The instruments used in this study detected that massage therapy caused no harm to the MS patients and a limited amount of improvement in their overall health was observed. As noted by the patients' personal rating of their health as well as their final comments, there appears to be an improvement to patient well-being due to the massage therapy. It is difficult to determine whether these improvements were due to the social interaction experienced during massage or a direct effect of the mechanisms underlying pain control or if the effect was a combination of the two. Regardless, improvement in the quality of life experienced by an individual with MS is of great importance and therefore further research is warranted.

## Figures and Tables

**Table 1:** Participant characteristics.

Participant characteristics (*n* = 24)	
Average age (years)	50 (30 to 72)
Gender (m : f)	9 : 17
Height range	1.56 meters to 1.95 meters
Average height	1.67 meters
Weight range	51 kg to 141 kg
Average weight	83.8 kg
EDSS* score range	3.5 to 6.5
Average EDSS* score	4.5
Time since initial MS diagnosis	10 months to 44 years
Previous use of massage prior to the study (yes : never)	18 : 6

*Kurtzke expanded disability status scale.

**Table 2:** Six-Minute Walk Test results. Lower severity group is individuals with an EDSS of 3.0–4.9, while higher severity group is individuals with an EDSS of 5.0–7.0.

Group	Group 1							
	Assessment 1 (A1)		Assessment 2 (A2)		Assessment 3 (A3)		Effect size	Effect size
	M	(SD)	M	(SD)	M	(SD)	A1 − A2	A2 − A3
Lower severity (*N* = 8)	489.25	(89.68)	512.75	(92.59)	499.63	(107.06)	−.26	.13
Higher severity (*N* = 5)	323.00	(131.92)	362.00	(148.55)	352.40	(144.16)	−.28	.04
Group 1 total (*N* = 13)	**425.31**	**(132.59)**	**454.77**	**(134.85)**	**443.00**	**(138.46)**	−**.22**	**.09**

Group	Group 2							
	Assessment 1		Assessment 2		Assessment 3		Effect size	Effect size
	M	(SD)	M	(SD)	M	(SD)	A1 − A2	A2 − A3

Lower severity (*N* = 4)	522.25	(42.25)	528.50	(40.81)	526.50	(52.91)	−.15	.04
Higher severity (*N* = 7)	240.00	(119.91)	244.29	(101.83)	248.14	(91.74)	−.03	**−**.04
Group 2 total (*N* = 11)	**342.64**	**(171.61)**	**347.64**	**(165.17)**	**349.36**	**(160.04)**	−**.03**	**−.01**

**Table 3:** Hamburg Quality of Life in MS results. The average score of the five subscales was weighed evenly to calculate the HAQUAMS averages used in analysis. Lower scores reflect more positive ratings.

Group	Group 1							
	Assessment 1		Assessment 2		Assessment 3		Effect size	Effect size
	M	(SD)	M	(SD)	M	(SD)	A1 − A2	A2 − A3
Lower severity (*N* = 8)	73.25	(17.20)	72.88	(16.37)	71.75	(13.49)	.02	.08
Higher severity (*N* = 5)	91.20	(22.04)	82.80	(22.95)	86.40	(24.87)	.37	−.15
Group 1 total (*N* = 13)	**80.15**	**(20.42)**	**76.69**	**(18.91)**	**77.38**	**(19.16)**	**.18**	−**.04**

Group	Group 2							
	Assessment 1		Assessment 2		Assessment 3		Effect size	Effect size
	M	(SD)	M	(SD)	M	(SD)	A1 − A2	A2 − A3

Lower severity (*N* = 4)	72.25	(30.08)	74.25	(33.25)	71.50	(21.89)	−.06	.10
Higher severity (*N* = 7)	82.86	(12.64)	83.71	(13.23)	83.14	(13.09)	−.07	.04
Group 2 total (*N* = 11)	**80.09**	**(19.55)**	**80.27**	**(21.44)**	**78.91**	**(17.73)**	−**.01**	**.07**

**Table 4:** Personal health rating. Lower scores reflect more positive ratings.

Group	Group 1							
	Assessment 1		Assessment 2		Assessment 3		Effect size	Effect size
	M	(SD)	M	(SD)	M	(SD)	A1 − A2	A2 − A3
Lower severity (*N* = 8)	2.88	(.83)	3.38	(.74)	3.38	(1.06)	−.64	0
Higher severity (*N* = 5)	4.00	(1.41)	4.00	(1.41)	4.40	(.89)	0	−.33
Group 1 total (*N* = 13)	**3.31**	**(1.18)**	**3.62**	**(1.04)**	**3.77**	**(1.09)**	−**.28**	−**.14**

Group	Group 2							
	Assessment 1		Assessment 2		Assessment 3		Effect size	Effect size
	M	(SD)	M	(SD)	M	(SD)	A1 − A2	A2 − A3

Lower severity (*N* = 4)	3.50	(1.00)	4.00	(.82)	3.00	(.82)	−.55	1.22
Higher severity (*N* = 7)	4.14	(.90)	4.29	(.49)	3.71	(.76)	−.21	.90
Group 2 total (*N* = 11)	**3.91**	**(.94)**	**4.18**	**(.60)**	**3.45**	**(.82)**	−**.34**	1.02*

*Statistically significant *P* < 0.05.
